# Effects of Exercise Preconditioning on Doxorubicin-Induced Liver and Kidney Toxicity in Male and Female Rats

**DOI:** 10.3390/ijms241210222

**Published:** 2023-06-16

**Authors:** Franccesco P. Boeno, Jay Patel, Ryan N. Montalvo, Stephanie S. Lapierre-Nguyen, Claire M. Schreiber, Ashley J. Smuder

**Affiliations:** Department of Applied Physiology & Kinesiology, University of Florida, Gainesville, FL 32608, USA; boeno.f@ufl.edu (F.P.B.); jpatel6@ufl.edu (J.P.); ryan.montalvo@ufl.edu (R.N.M.); slapierre@ufl.edu (S.S.L.-N.); claire.schreiber@ufl.edu (C.M.S.)

**Keywords:** hepatotoxicity, nephrotoxicity, anthracycline, exercise preconditioning

## Abstract

Doxorubicin (DOX) is a highly effective chemotherapy agent prescribed for cancer treatment. However, the clinical use of DOX is limited due to off-target toxicity in healthy tissues. In this regard, hepatic and renal metabolic clearance results in DOX accumulation within these organ systems. Within the liver and kidneys, DOX causes inflammation and oxidative stress, which promotes cytotoxic cellular signaling. While there is currently no standard of care to treat DOX hepatic- and nephrotoxicity, endurance exercise preconditioning may be an effective intervention to prevent elevations in liver alanine transaminase (ALT) and aspartate aminotransferase (AST) and to improve kidney creatinine clearance. To determine whether exercise preconditioning is sufficient to reduce liver and kidney toxicity resulting from acute exposure to DOX chemotherapy treatment, male and female Sprague–Dawley rats remained sedentary or were exercise trained prior to saline or DOX exposure. Our findings demonstrate that DOX treatment elevated AST and AST/ALT in male rats, with no effects of exercise preconditioning to prevent these increases. We also showed increased plasma markers of renin–angiotensin–aldosterone system (RAAS) activation and urine markers of proteinuria and proximal tubule damage, with male rats revealing greater differences compared to females. Exercise preconditioning showed improved urine creatinine clearance and reduced cystatin c in males, while females had reduced plasma angiotensin II (AngII) levels. Our results demonstrate both tissue- and sex-specific responses related to the effects of exercise preconditioning and DOX treatment on markers of liver and kidney toxicity.

## 1. Introduction

Doxorubicin (DOX) is a first-line chemotherapy agent used to treat solid tumor and hematological cancers [1,2]. DOX induces cancer cell death by destabilizing DNA and disrupting replication and transcription and by causing cellular damage through the generation of oxygen radicals [3]. Although DOX is a highly effective antineoplastic agent, its clinical use is limited due to multi-organ toxic effects. Following administration, DOX is rapidly cleared from the circulation and preferentially localizes within the liver and kidneys [4]. In addition to its disposition within these tissues, the hepatic and renal systems play a primary role in the metabolism and excretion of DOX [5,6]. Specifically, it is estimated that greater than 50% of DOX is excreted by the hepatobiliary pathway within 7 days of administration and ~12% is excreted in the urine, with the greatest portion retaining its original structure or metabolized to doxorubicinol [7,8,9].

Drug-induced liver injury resulting from chemotherapy administration has been confirmed using the Roussel Uclaf Causality Assessment Method (RUCAM) where elevated circulating liver enzymes (i.e., cystatin c, albumin, aspartate aminotransferase, etc.) are associated with greater risk of hepatotoxicity [10,11]. Kidney toxicity was also established as early as 1977 via a case study where DOX chemotherapy was associated with renal failure [12]. The development of these toxicities requires greater understanding as off-target organ dysfunction often delays or prevents continuation of cancer treatment [13].

While the exact mechanisms of DOX hepato- and nephrotoxicity remain unknown, it is hypothesized that accumulation and metabolism of DOX within these organ systems results in free radical damage, lipid peroxidation and apoptosis [14,15]. Specifically, in the liver, literature shows that reactive oxygen species (ROS) are formed via redox cycling of the DOX semiquinone or through NADPH oxidase [16], whereas kidney oxidative damage likely occurs through the formation of a highly reactive iron-DOX complex [17,18]. In both organs, altered antioxidant levels, mitochondrial damage and inflammation contribute to tissue pathology [14,19,20].

The off-target toxicity of DOX to multiple organ systems necessitates a systemic therapy that can preserve its anti-tumor efficacy while limiting its harmful effects. However, there are currently no clinical therapies approved for the prevention or treatment of DOX liver and kidney toxicity. In this regard, aerobic exercise has been hypothesized to modify DOX cytotoxic signaling to reduce oxidative damage and inflammation [2]. To date, limited work has been done focusing on the cytoprotective effects of exercise on these tissues. Therefore, this study utilized a two-week exercise preconditioning intervention to determine whether exercise prior to DOX exposure can elicit a phenotype that protects against hepato- and nephrotoxicity. In addition, we determined the sex-specific responses in the development of liver and kidney toxicity and investigated the signaling pathways affected by exercise.

## 2. Results

### 2.1. General Effects of Exercise Preconditoning and DOX

All exercise-preconditioned rats completed the full ten days of training without incident. Compared to sedentary (SED) rats, 10 days of exercise training (EX) resulted in a significant reduction (*p* < 0.05) in body weight in males (SED = 365.4 ± 6.3 g; EX = 395.5 ± 6.3 g), whereas no difference in body weight was seen in females following the exercise preconditioning protocol (SED = 244.6 ± 2.8 g; EX = 242.8 ± 3.7 g). Final body weight taken two days following saline or DOX treatment showed a significant main effect (*p* < 0.05) of both EX and DOX in males (SED-SALINE: 398.6 ± 8.0 g; EX-SALINE: 375.9 ± 9.8 g; SED-DOX: 367.4 ± 8.7 g; EX-DOX: 375.9 ± 9.8 g). No effects of treatment were seen between groups for the females (SED-SALINE: 239.6 ± 7.2 g; EX-SALINE: 246.3 ± 5.0 g; SED-DOX: 236.3 ± 3.9 g; EX-DOX: 239.0 ± 5.3 g).

### 2.2. Preconditioning Exercise Alters Markers of Kidney Toxicity

**Figure 1:**
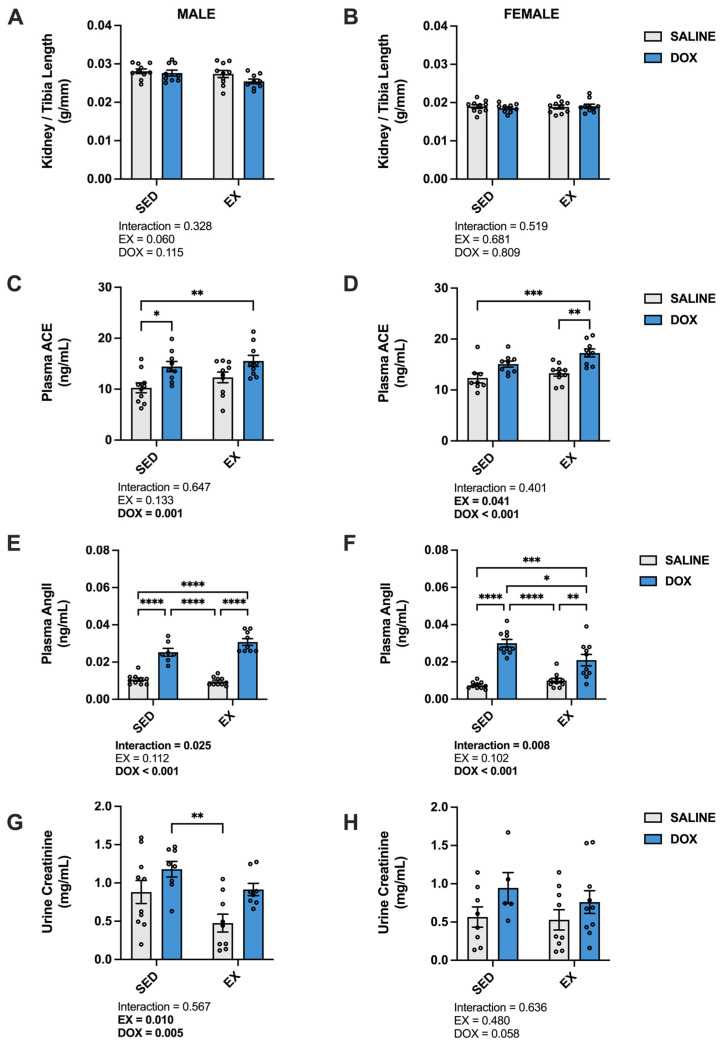
(**A**,**B**) Kidney/tibia length, (**C**,**D**) plasma levels of angiotensin converting enzyme (ACE), (**E**,**F**) plasma levels of angiotensin II (AngII), and (**G**,**H**) levels of urine creatinine from sedentary (SED) or exercise-preconditioned (EX) male (left) and female (right) rats treated with doxorubicin (DOX) or saline. Data are presented as mean ± SEM. Two-way ANOVA differences are indicated below the graphs. Circles indicate individual data points. * = significant difference between groups (*p* < 0.05), ** = significant difference between groups (*p* < 0.01), *** = significant difference between groups (*p* < 0.001), **** = significant difference between groups (*p* < 0.0001).

Changes in kidney weight, circulating components of renin–angiotensin–aldosterone system (RAAS) signaling and urine creatinine are all markers associated with DOX nephrotoxicity [14,21,22,23]. In this study, kidney/tibia length did not differ between groups for either males or females. Absolute kidney weight in males showed a significant main effect of EX (*p* < 0.05) (SED-SALINE: 1.20 ± 0.03 g; EX-SALINE: 1.14 ± 0.04 g; SED-DOX: 1.15 ± 0.03 g; EX-DOX: 1.06 ± 0.03 g), while no difference existed in absolute kidney weight for females (SED-SALINE: 0.73 ± 0.02 g; EX-SALINE: 0.73 ± 0.02 g; SED-DOX: 0.72 ± 0.02 g; EX-DOX: 0.73 ± 0.02 g). Plasma levels of angiotensin-converting enzyme (ACE) and angiotensin II (AngII) and urine levels of creatine were all affected by DOX exposure (Figure 1). Specifically, measurement of ACE revealed main effects of DOX treatment in both sexes, and a main effect of exercise in females. Multiple comparisons showed a significant increase in SED-DOX and EX-DOX compared to SED-SALINE (*p* < 0.05) in males, and a significant increase in ACE in EX-DOX compared to SED-SALINE and EX-SALINE in females (*p* < 0.05). AngII also showed a main effect of DOX for both sexes. In males, multiple comparisons showed that AngII was significantly elevated in the plasma of SED-DOX and EX-DOX rats compared to SED-SALINE and EX-SALINE. In females, plasma AngII was significantly elevated in SED-DOX rats compared to all other groups and in EX-DOX rats compared to SED-SALINE and EX-SALINE. Main effects of both DOX and exercise were present in males for urine creatine, with SED-DOX significantly elevated compared to EX-SALINE (*p* < 0.05).

**Figure 2:**
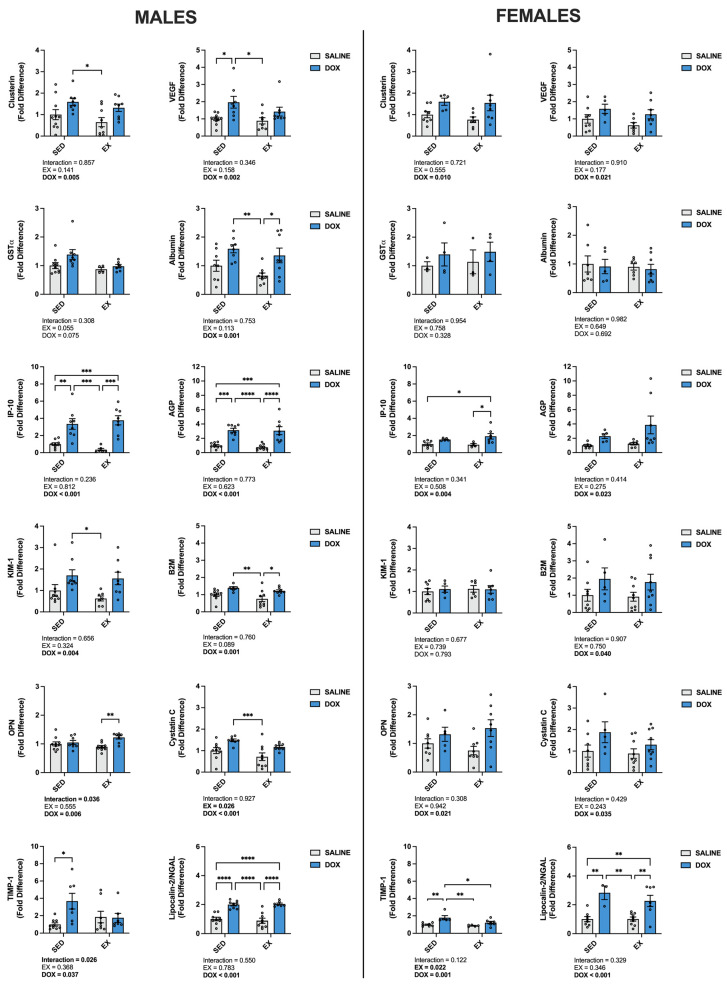
Urine levels of clusterin, α-glutathione-S-transferases (GST-α), IFN-gamma-inducible protein 10 (IP-10), kidney injury molecule-1 (KIM-1), osteopontin (OPN), tissue inhibitor matrix metalloproteinase 1 (TIMP-1), vascular endothelial growth factor (VEGF), albumin, α-1 acid glycoprotein (AGP), β-2-microglobulin (B2M), cystatin C and neutrophil gelatinase-associated lipocalin (Lipocalin-2/NGAL). Data are presented as mean ± SEM. Two-way ANOVA differences are indicated below the graphs. Circles indicate individual data points. * = significant difference between groups (*p* < 0.05), ** = significant difference between groups (*p* < 0.01), *** = significant difference between groups (*p* < 0.001), **** = significant difference between groups (*p* < 0.0001).

Multiplex analysis of urine from each treatment group was also performed to determine changes to markers of nephrotoxicity (Figure 2). For males, a main effect of DOX existed for clusterin, IFN-gamma-inducible protein 10 (IP-10), kidney-injury molecule-1 (KIM-1), osteopontin (OPN), tissue inhibitor matrix metalloproteinase-1 (TIMP-1), vascular endothelial growth factor (VEGF), albumin, α-1 acid glycoprotein (AGP), β-2-microglobulin (B2M), cystatin c and neutrophil gelatinase-associated lipocalin (Lipocalin-2/NGAL) (*p* < 0.05). Cystatin c also showed a main effect of EX (*p* < 0.05). In addition, multiple comparisons revealed a significant increase in IP-10, AGP and Lipocalin-2/NGAL in SED-DOX and EX-DOX male rats compared to SED-SALINE and EX-SALINE (*p* < 0.05). SED-DOX was significantly elevated for clusterin, KIM-1 and cystatin c compared to EX-SALINE (*p* < 0.05). SED-DOX and EX-DOX had higher levels of urine albumin and B2M compared to EX-SALINE (*p* < 0.05). In addition, OPN was elevated in EX-DOX compared to EX-SALINE, TIMP-1 was elevated in SED-DOX compared to SED-SALINE and VEGF was elevated in SED-DOX compared to SED-SALINE and EX-SALINE (*p* < 0.05). Urine from female rats showed main effects of DOX for clusterin, IP-10, OPN, TIMP-1, VEGF, AGP, B2M, cystatin c and Lipocalin-2/NGAL. Main effects of EX were only seen for TIMP-1. Multiple comparisons showed a significant increase in urine IP-10 in EX-DOX compared to SED-SALINE and EX-SALINE, in TIMP-1 in SED-DOX compared to all other groups and in Lipocalin-2/NGAL in SED-DOX and EX-DOX compared to both saline-treated control groups (*p* < 0.05).

### 2.3. Mechanisms of Exercise Protection against DOX Nephrotoxicity

**Figure 3:**
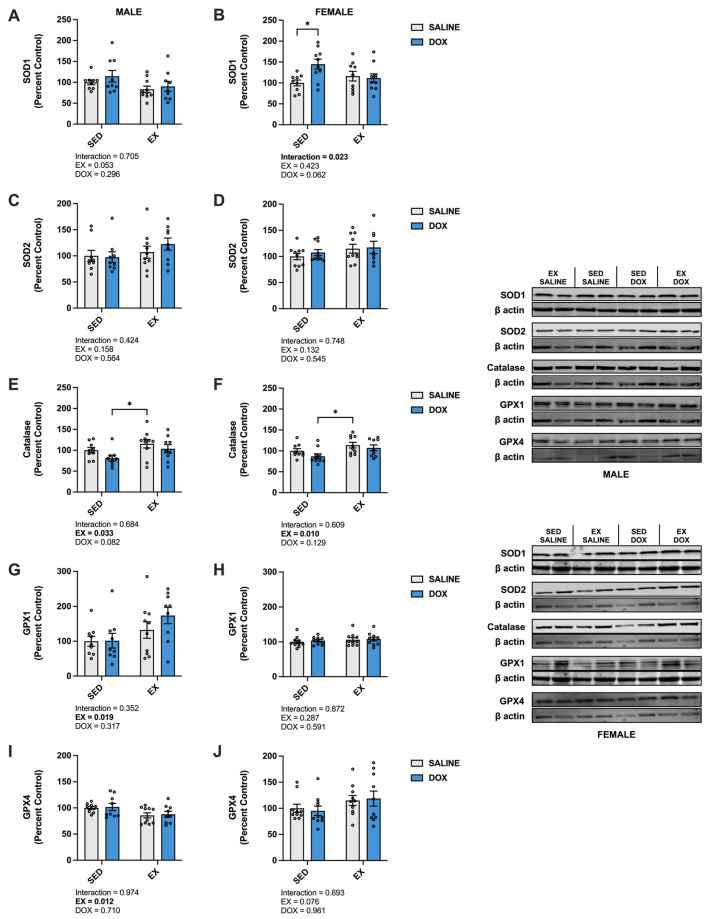
Kidney protein expression of (**A**,**B**) superoxide dismutase (SOD) 1, (**C**,**D**) SOD2, (**E**,**F**) catalase, (**G**,**H**) glutathione peroxidase (GPX) 1, and (**I**,**J**) GPX4 primary antioxidants from sedentary (SED) or exercise-preconditioned (EX) male (left) and female (right) rats treated with doxorubicin (DOX) or saline. Data are presented as mean ± SEM. Representative western blots are shown to the right of the graphs. Two-way ANOVA differences are indicated below the graphs. Circles indicate individual data points. * = significant difference between groups (*p* < 0.05).

**Figure 4:**
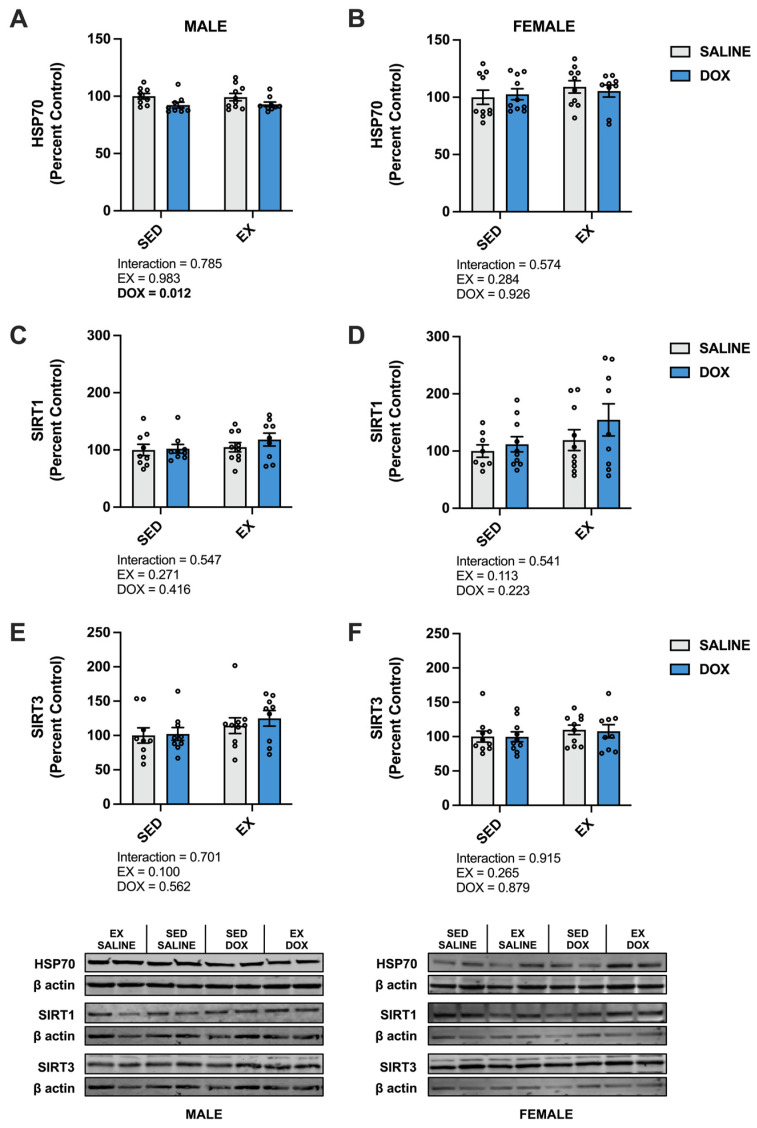
Kidney protein expression of (**A**,**B**) heat shock protein 70 (HSP70), (**C**,**D**) sirtuin (SIRT) 1, and (**E**,**F**) SIRT3 from sedentary (SED) or exercise-preconditioned (EX) male (left) and female (right) rats treated with doxorubicin (DOX) or saline. Data are presented as mean ± SEM. Representative western blots are shown below the graphs. Two-way ANOVA differences are indicated below the graphs. Circles indicate individual data points.

Kidney protein expression of superoxide dismutase (SOD) 1 did not differ between groups for males. Females showed a significant increase in SOD1 in the SED-SALINE rats compared to the SED-DOX rats (*p* < 0.05). No difference existed between groups for SOD2 for either sex. A significant main effect of exercise was seen for catalase expression in both male and female rats, with EX-SALINE catalase expression significantly greater than SED-DOX expression (*p* < 0.05). Measurement of glutathione peroxidase (GPX) 1 and GPX4 revealed a significant main effect of exercise but only in males (Figure 3). The expression of heat shock protein 70 (HSP70) showed a main effect of DOX treatment in males and no differences between groups for females. Finally, sirtuin (SIRT) 1 and SIRT3 expression was not affected by either DOX or exercise for either sex (Figure 4).

### 2.4. Preconditioning Exercise Alters Markers of Liver Toxicity

**Figure 5:**
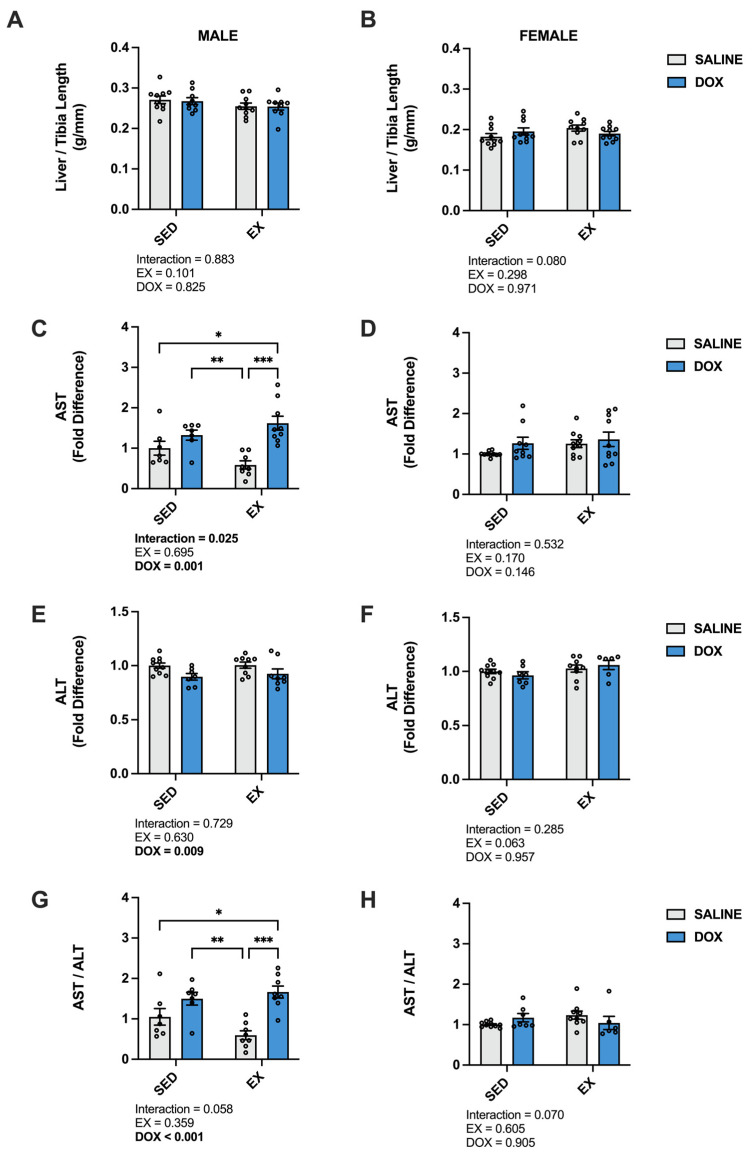
(**A**,**B**) Liver/tibia ratio, (**C**,**D**) plasma levels of aspartate aminotransferase (AST), (**E**,**F**) plasma levels of alanine transaminase (ALT), and (**G**,**H**) ratio of AST/ALT from sedentary (SED) or exercise-preconditioned (EX) male (left) and female (right) rats treated with doxorubicin (DOX) or saline. Data are presented as mean ± SEM. Two-way ANOVA differences are indicated below the graphs. Circles indicate individual data points. * = significant difference between groups (*p* < 0.05). ** = significant difference between groups (*p* < 0.01), *** = significant difference between groups (*p* < 0.001).

Liver damage was evaluated by calculating the liver to tibia length ratio and measuring the circulating levels of aspartate aminotransferase (AST) and alanine transaminase (ALT) [24]. Our results showed no effect of DOX or exercise on the ratio of liver weight to tibia length. In addition, no differences were seen when comparing absolute liver weight for males (SED-SALINE: 11.5 ± 0.43 g; EX-SALINE: 10.7 ± 0.36 g; SED-DOX: 11.5 ± 0.43 g; EX-DOX: 10.6 ± 0.38 g) or females (SED-SALINE: 7.01 ± 0.30 g; EX-SALINE: 7.87 ± 0.30 g; SED-DOX: 7.55 ± 0.35 g; EX-DOX: 7.30 ± 0.21 g). There was a significant main effect of DOX to elevate AST and reduce ALT in the plasma of male rats. These shifts resulted in an overall significant main effect of DOX to elevate the ratio of AST to ALT in the male rats. Multiple comparisons revealed a significant increase in AST and AST/ALT in SED-DOX compared to EX-SALINE and in EX-DOX compared to SED-SALINE and EX-SALINE (*p* < 0.05). No significant differences were evident in these enzyme markers of hepatotoxicity in females (Figure 5).

### 2.5. Mechanisms of Exercise Protection against DOX Hepatotoxicity

**Figure 6:**
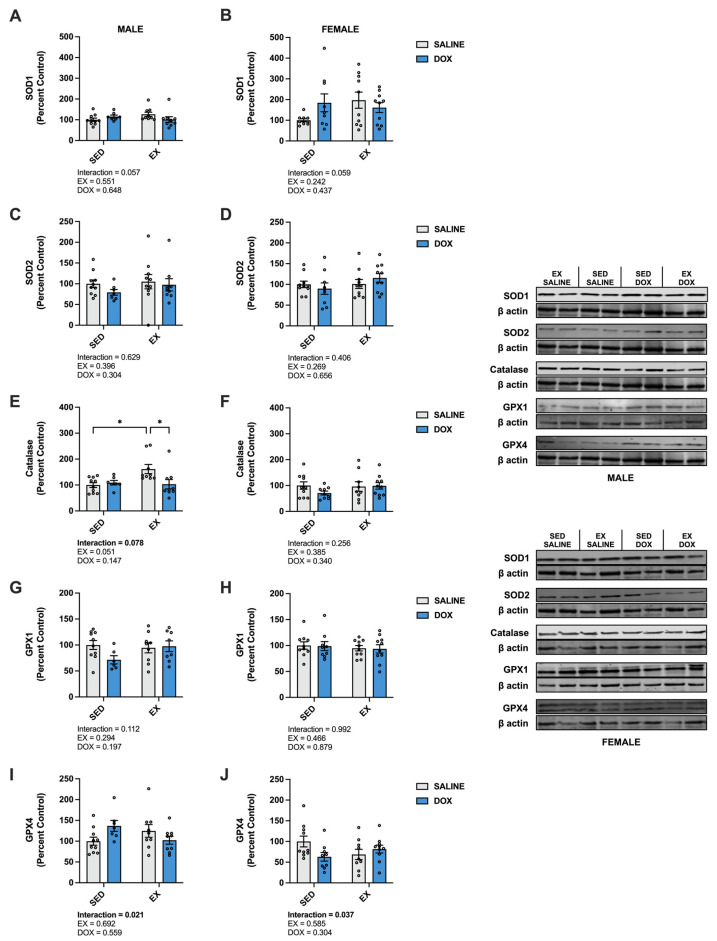
Liver protein expression of (**A**,**B**) superoxide dismutase (SOD) 1, (**C**,**D**) SOD2, (**E**,**F**) catalase, (**G**,**H**) glutathione peroxidase (GPX) 1, and (**I**,**J**) GPX4 primary antioxidants from sedentary (SED) or exercise-preconditioned (EX) male (left) and female (right) rats treated with doxorubicin (DOX) or saline. Data are presented as mean ± SEM. Representative western blots are shown to the right of the graphs. Two-way ANOVA differences are indicated below the graphs. Circles indicate individual data points. * = significant difference between groups (*p* < 0.05).

**Figure 7:**
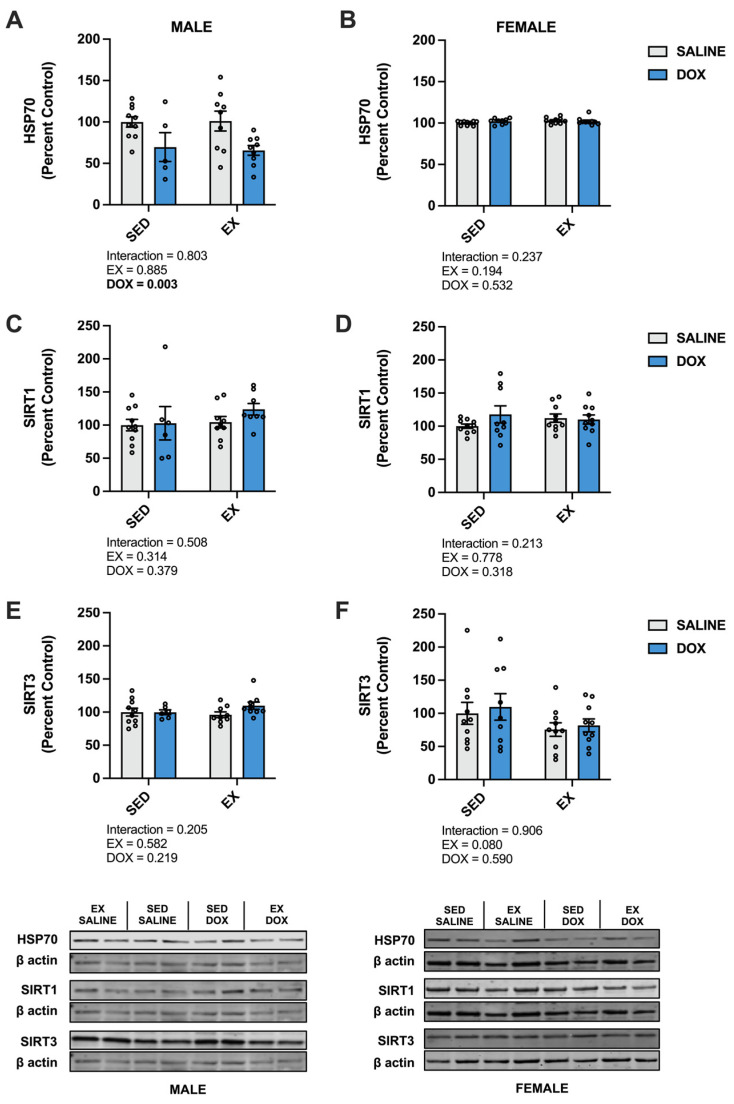
Liver protein expression of (**A**,**B**) heat shock protein 70 (HSP70), (**C**,**D**) sirtuin (SIRT) 1, and (**E**,**F**) SIRT3 from sedentary (SED) or exercise-preconditioned (EX) male (left) and female (right) rats treated with doxorubicin (DOX) or saline. Data are presented as mean ± SEM. Representative western blots are shown below the graphs. Two-way ANOVA differences are indicated below the graphs. Circles indicate individual data points.

In the liver, there was a significant increase in catalase in male EX-SALINE rats compared to SED-SALINE and EX-DOX (*p* < 0.05), with no effect of treatment or activity in the females. No differences were discerned between groups for SOD1, SOD2, GPX1 or GPX4 protein expression for either sex (Figure 6). In males, HSP70 displayed a significant main effect for DOX treatment to reduce protein expression with no effect for the females. No differences were found in the hepatic protein expression of SIRT1 or SIRT3 for males or females (Figure 7).

## 3. Discussion

DOX is a widely utilized and highly effective antineoplastic agent. However, multi-organ toxicity severely limits its use [2,9]. The renal and hepatic systems are particularly affected due to their role in metabolism and clearance of DOX. Specifically, the kidneys and liver have a high affinity for DOX, allowing for preferential accumulation in these organs following systemic administration [6]. Although there is currently no standard of care to prevent DOX toxicity in healthy organs, exercise can protect against cardiac and skeletal muscle toxicity via redox-related mechanisms [5,16]. Therefore, utilizing a preclinical model, we investigated the effects of aerobic exercise preconditioning on markers of DOX-induced nephro- and hepatotoxicity. A detailed discussion of our findings follows.

### 3.1. DOX-Induced Nephrotoxicity and Exercise Preconditioning

DOX-induced damage to the kidneys results from the accumulation of potassium, phosphate and uric acid as a result of tumor lysis and unaltered DOX and its metabolites [25]. Independent of tumor breakdown products, DOX metabolism and excretion through the urine over several days induces renal inflammation and oxidative damage [14,26]. Activation of this pro-inflammatory phenotype was evident in the kidneys of our DOX-treated rats as IP-10, OPN and lipocalin-2 were all elevated in the urine of both male and female rats. Additional markers of renal dysfunction elevated in the urine of both male and female rats included creatinine, TIMP-1, VEGF, B2M and cystatin c. These markers increase in the urine as a result of both tubular damage and glomerulopathy [27,28]. Sex-specific responses differed when comparing the number of proteins elevated in the urine of DOX-treated rats. Clusterin, KIM-1, albumin and AGP were only elevated in males. These differences suggest that males may be more susceptible to renal dysfunction following DOX treatment. Circulating markers of the RAAS are also associated with renal failure, and both ACE and AngII were elevated in the plasma of male and female DOX-treated rats [22]. Studies have shown that ROS production can induce overactivation of the RAAS, which plays a role in the pathogenesis of renal toxicity, and administration of ACE inhibitors and angiotensin receptor blockers provides therapeutic benefit [21,23].

Limited studies have addressed the potential benefit of exercise to combat DOX nephrotoxicity. However, existing data support the postulate that exercise training provides beneficial adaptations to reduce urine and plasma biomarkers of renal toxicity and improve histological markers of damage [29,30,31,32,33]. Our results support an exercise effect to reduce the level of circulating AngII in females only, urine creatinine and cystatin c in males only and urine TIMP-1 in both males and females. Mechanisms for these reductions are potentially related to reductions in oxidative damage to the kidneys via an exercise-induced increase in catalase expression in both sexes and GPX1 in the males.

### 3.2. DOX-Induced Hepatotoxicity and Exercise Preconditioning

DOX metabolism in the liver is associated with the production of ROS, inflammation and mitochondrial dysfunction [20,24]. Specific localization within liver mitochondria results in oxidative damage, reduced oxidative phosphorylation and impaired ATP production [34]. This reduces the hepatocytes’ ability to perform energy-dependent processes required for DOX detoxification and elimination, and promotes cellular apoptosis [35,36]. Hepatocellular death results in the leakage of enzymes into circulation, and thus, liver dysfunction can be assessed via the quantification of these biomarkers [15,37]. Specifically, elevated circulating AST and ALT and an AST/ALT ratio greater than 1 are indicative of hepatic damage [6]. Similar to previous reports, our results demonstrated a significant effect of DOX administration to increase plasma levels of AST [38]. In contrast, we show an effect of DOX to reduce ALT; however, the resulting AST/ALT ratio was elevated above 1 in both the sedentary and exercise-trained DOX-treated groups, representing overall hepatic impairment. Interestingly, this affect was only seen in the male rats, with no difference between groups for AST, ALT or AST/ALT in females. Increasing work is needed to understand these sex-specific responses as data suggest that DOX clearance is slower in females compared to males and that females have greater susceptibility for drug-induced liver injury [39,40].

While liver dysfunction is established in both clinical and preclinical evaluation of DOX toxicity, no therapy exists to reduce this adverse effect. Exercise training has been evaluated for benefits to other organ systems in patients receiving DOX chemotherapy [2]. However, no clinical report has evaluated whether exercise elicits beneficial effects to protect against liver toxicity. Studies in rodents have shown improvements in mitochondrial function, markers of oxidative stress and inflammation [20,41,42]. Only two studies to date have evaluated the effects of exercise on AST and ALT in animals treated with DOX [43,44]. In these studies, 12 weeks of treadmill exercise preconditioning or 5 weeks of strength training concomitant to DOX treatment showed a reduction in circulating AST. Interestingly, ALT was only reduced with the aerobic preconditioning exercise [44], while it was increased with strength training [43]. These studies were performed in male rodents and support our findings that markers of hepatotoxicity are increased in male rodents treated with DOX. The difference in the exercise effects on circulating markers between the three distinct exercise training protocols highlights the need for additional research to determine the optimal exercise prescription for the prevention of DOX-induced liver dysfunction.

## 4. Materials and Methods

### 4.1. Experimental Animals 

The University of Florida (UF) Institutional Animal Care and Use Committee (IACUC approval #202011110) approved these experiments. This study was performed in accordance with the National Institutes of Health Guide for the Care and Use of Laboratory Animals. Male and female Sprague–Dawley rats (4–5 months old) were used in these experiments and were obtained from Charles River Laboratories (Wilmington, MA, USA). Rats were housed in pairs in the UF Animal Care Services vivarium. They were maintained on a 12:12 light dark cycle, monitored daily and provided rodent diet 2918 (Envigo, Indianapolis, IN, USA) and water ad libitum.

### 4.2. Study Design

Rats were randomly assigned to sedentary (SED) or exercise preconditioning (EX) groups. Exercise preconditioning consisted of five days of habituation to treadmill running (30 m/min, 0% grade, 10, 20, 30, 40, 50 min on days 1–5). Following habituation, rats rested for two days prior to initiation of a 10-day training protocol [45]. Each training session was performed at 30 m/min, 0% grade for 60 min. Twenty-four hours after the last training session or at an equal time for sedentary rats, each group was further divided to receive either DOX (20 mg/kg I.P.) (Teva Pharmaceuticals, Parsippany, NJ, USA) or saline (equal volumes to DOX) [45,46]. Forty-eight hours following DOX/saline exposure, rats were euthanized. Blood was collected via K_3_EDTA tubes (454217, Greiner, Kremsmünster, Austria), and plasma was separated using centrifugation at 500× *g* for 10 min at 4 °C. Urine was collected using cytocentesis. The liver and kidneys were removed and weighed prior to flash freezing in liquid nitrogen.

A sample size of *n* = 10/group was used to determine if differences existed between groups. This was chosen based on our previous experience with the experimental model [20,45,46,47]. Prior to study initiation, there were no differences in body weight between groups for males or females (Males—SED-SALINE: 332.0 ± 10.3 g, EX-SALINE: 330.3 ± 10.3 g, SED-DOX: 330.8 ± 4.8 g, EX-DOX: 329.8 ± 6.0 g; Females—SED-SALINE: 224.8 ± 3.6 g, EX-SALINE: 224.8 ± 4.2 g, SED-DOX: 224.2 ± 3.6 g, EX-DOX: 222.5 ± 3.3 g). No rats died as a result of any treatment. Two male rats, one SED-DOX and one EX-DOX, were removed from the study due to misinjection.

### 4.3. Kidney Toxicity

Urine was centrifuged at 13,000 rpm for 10 min at 4 °C and assayed via Milliplex Rat Kidney Toxicity Panel 1 (RKTX1MAG-37K; clusterin, GSTα, IP-10, KIM-1, OPN, TIMP-1 and VEGF) and Milliplex Rat Kidney Toxicity Panel 2 (RKTX1MAG-37K; albumin, AGP, B2M, Cystatin C, EGF, Lipocalin-2/NGAL) (EMD Millipore Corporation, Billerica, MA, USA) following the manufacturer’s instructions. Urine creatinine levels were quantified via a Creatinine Urinary Detection Kit (EIACUN, ThermoFisher, Waltham, MA, USA) following the manufacturers’ instructions and by a blinded researcher. Values below the lower limit of quantification (LLOQ) were removed from the analysis.

### 4.4. Plasma Analysis 

Plasma levels of ACE (MBS2020292, MyBioSource, San Diego, CA, USA), AngII (CSB-E04494r, Cusabio, Houston, TX, USA), ALT (ab234579, Abcam, Waltham, MA, USA) and AST (ab263883, Abcam, Waltham, MA, USA) were measured via ELISA according to manufacturer’s instructions by a blinded researcher.

### 4.5. Western Blot Analysis

Liver and kidney tissue were homogenized 1:10 (wt/vol) in 5 mM Tris (pH 7.5) and 5 mM EDTA (pH 8.0) with a protease inhibitor cocktail (P8340, Sigma-Aldrich, St. Louis, MO, USA) and centrifuged at 1500× *g* for 10 min at 4 °C. Supernatant was separated from the pellet, and supernatant protein content was assessed using the Bradford method (B6916, Sigma-Aldrich, St. Louis, MO, USA). A total of 30–40 µg of protein was separated on 4–20% precast gels (5671095, Bio-Rad, Hercules, CA, USA) and transferred to nitrocellulose membranes (1620112, Bio-Rad, Hercules, CA, USA), followed by blocking in 5% non-fat milk, washing in PBST and a minimum of one overnight incubation at 4 °C with primary antibodies directed against SOD1 (1;1:000; sc-11407), HSP70 (1:000; sc-32239) (Santa Cruz Biotechnology, Dallas, TX, USA), SOD2 (1:1000; ab68155), catalase (1:000; ab52477), GPX1 (1:000; ab22604), GPX4 (1:1000; ab125066) (Abcam, Waltham, MA, USA), SIRT1 (1:000; 8469S), β-actin (1:500; #4967) (Cell Signaling Technologies, Danvers, MA, USA) and SIRT3 (1:100; 10099-1-AP) (Proteintech, Rosemont, IL, USA) diluted 1:1 in Odyssey blocking buffer (LI-COR Biosciences, Lincoln, NE, USA) and PBS. Membranes were exposed to rabbit AlexaFluor 680 IgG or 800 IgG (LI-COR) secondary antibodies. Imaging and analysis were performed using the Odyssey CLx imaging system and Image Studio software v5.4 (LI-COR). Western blots were loaded in random order and performed by a researcher blinded to the experimental groups.

### 4.6. Statistical Analysis

All data was tested for normal distribution, followed by square root transformation on data sets that did not meet this assumption. Comparison between groups were determined via two-way analysis of variance (ANOVA) to determine whether main effects existed for treatment and activity. When significant interactions were present, a Tukey multiple comparisons test was performed post hoc. Significance was established at *p* < 0.05. Data are presented as mean ± standard error.

## 5. Conclusions

These data demonstrate that DOX-induced damage to the liver and kidneys resulted in increased RAAS system activation and circulating levels of pro-inflammatory proteins. While previous work has shown that exercise preconditioning may prevent DOX tissue toxicity by increasing antioxidant capacity, HSP70 and SIRT1/3, our data show limited effects of exercise to upregulate the expression of these proteins. Additional work is needed to determine the precise mechanisms by which DOX elicits hepato- and nephrotoxicity and the optimal exercise prescription to confer protection.

## Data Availability

The authors confirm that the data supporting the findings of this study are available within the article and/or are available upon reasonable request, and further questions can be directed to the corresponding author.
